# Machine learning approach for the development of a crucial tool in suicide prevention: The Suicide Crisis Inventory-2 (SCI-2) Short Form

**DOI:** 10.1371/journal.pone.0299048

**Published:** 2024-05-10

**Authors:** Gabriele P. De Luca, Neelang Parghi, Rawad El Hayek, Sarah Bloch-Elkouby, Devon Peterkin, Amber Wolfe, Megan L. Rogers, Igor Galynker

**Affiliations:** 1 Department of Psychiatry, Faculty of Medicine and Psychology, University of Rome Sapienza, Rome, Italy; 2 Department of Biology, New York University, New York City, New York, United States of America; 3 Department of Psychiatry, Mount Sinai Beth Israel, New York City, New York, United States of America; 4 Icahn School of Medicine at Mount Sinai, New York City, New York, United States of America; 5 Department of Psychology, Texas State University, San Marcos, Texas, United States of America; University Kebangsaan Malaysia, MALAYSIA

## Abstract

The Suicide Crisis Syndrome (SCS) describes a suicidal mental state marked by entrapment, affective disturbance, loss of cognitive control, hyperarousal, and social withdrawal that has predictive capacity for near-term suicidal behavior. The Suicide Crisis Inventory-2 (SCI-2), a reliable clinical tool that assesses SCS, lacks a short form for use in clinical settings which we sought to address with statistical analysis. To address this need, a community sample of 10,357 participants responded to an anonymous survey after which predictive performance for suicidal ideation (SI) and SI with preparatory behavior (SI-P) was measured using logistic regression, random forest, and gradient boosting algorithms. Four-fold cross-validation was used to split the dataset in 1,000 iterations. We compared rankings to the SCI–Short Form to inform the short form of the SCI-2. Logistic regression performed best in every analysis. The SI results were used to build the SCI-2-Short Form (SCI-2-SF) utilizing the two top ranking items from each SCS criterion. SHAP analysis of the SCI-2 resulted in meaningful rankings of its items. The SCI-2-SF, derived from these rankings, will be tested for predictive validity and utility in future studies.

## Introduction

Suicide is a major global health concern that resulted in over 700,000 deaths worldwide in 2019 [1]. Despite all prevention efforts, suicide is the second leading cause of death in the United States among adolescents and young adults [2], and international suicide rates have increased 30% between 1996 and 2016 [3]. Of note, the coronavirus disease 2019 (COVID-19) has exacerbated this concern, as the pandemic has had negative impacts on psychopathology and associated suicide risk factors, such as financial strain [4–8]. Unemployment alone could lead to a three-fold increase in suicide risk [9]. However, other studies show that there has been a decrease in suicide risk especially during the first few months of the pandemic. Since current methods of identifying those at risk for imminent suicide are ineffective [10–13], the development of efficient and successful methods to preemptively identify at-risk individuals and prevent suicidal behavior is both urgent and essential.

Current suicide risk assessment methods are based on patients’ self-report of suicidal ideation (SI) [14–17]. However, literature on psychological autopsies of suicide decedents indicates that up to 75% never disclosed their SI to anyone [3, 18]. Although some individuals consciously try to hide their suicidal thoughts from their clinicians [19, 20] and are rather inclined to disclose such thoughts anonymously to researchers [21, 22]. SI also varies in intensity, meaning that others might not exhibit SI until minutes or hours before any suicidal behavior [23–26]. Additionally, an impulsive or subconsciously intentional suicide attempt could occur without any prior SI [27, 28]. Thus, new methods of identifying at-risk individuals for imminent suicide must not rely exclusively on SI.

### Assessment of short-term risk, Suicide Crisis Syndrome, and the development of the Suicide Crisis Inventory and Suicide Crisis Inventory–2

The Suicide Crisis Syndrome (SCS), originally labeled the Suicide Trigger State (STS) [29], is a suicidal, hyper-aroused, negative affective mental state that represents the las chain in a cascade of psychological processes described by the Narrative Crisis Model of Suicide [NCM; 30–32] does not include SI. The first factor analysis of the SCS revealed five factors. Factor one, “frantic hopelessness,” described an unescapable feeling of entrapment and carried more than 50% of the predictive power for near-term suicidal behavior. Factors two to four were emotional pain, ruminative flooding, panic/dissociation, fear of dying and carried less predictive value [29, 33, 34]. The SCS was initially assessed using the Suicide Crisis Inventory (SCI), a 49-item self-report psychometric measure primarily used in research. The SCI demonstrated good discriminant, convergent, predictive, and incremental validity over SI for short-term suicidal behavior [35, 36].

More recently, two additional symptom domains—overarousal and social withdrawal—were added to the SCI in order to improve its predictive validity for near-term suicidal behavior [37]. The revised SCI-2 demonstrated excellent predictive validity for near-term suicidal behavior [38–40] as well as excellent internal consistency and good convergent, discriminant, and current criterion validity [38]. In both factor and network analyses, the 61-item SCI-2 grouped into five factors (i.e., entrapment, affective disturbance, loss of cognitive control, hyperarousal, social withdrawal) and formed an interrelated network of symptoms with entrapment as its core component [38]. This revised form of the SCI is the template for the proposed suicide specific diagnosis of SCS [39, 41], currently under review by the American Psychiatric Association steering committee for inclusion in the Diagnostic and Statistical Manual (DSM).

In this context, the development of an SCI-2 short form (SCI-2-SF) is essential for clinical use, as a brief assessment tool provides multiple advantages. Fewer questions can reduce the burden felt by those administering the assessment, as there is less to review before obtaining important patient information. Similarly, long questionnaires are not conducive to fast-paced and intense clinical settings. Thus, health workers might appreciate the benefits of a brief tool. Additionally, shorter assessments help reduce dropout rates and ease the data entry process for EHR systems [42].

### Machine learning and Shapley Additive Explanation (SHAP) analysis

Machine learning (ML) is a computational strategy that automatically selects the most fitting methods and parameters to reach an optimal solution to a problem, rather than being programmed by a human a priori to deliver a fixed response. The computer actively “learns” how to carry out a particular task in the best possible way [43]. ML techniques have been successfully applied in many health-care settings and used in suicide risk analysis. Retrospective ML studies of large hospital databases showed greater predictive validity of suicidal behavior compared to ordinary methods [44, 45]. ML has also been employed in prospective studies. For example, it has been demonstrated that analyzing patients’ verbal and nonverbal suicide thought markers via ML could predict suicidal behavior with 85% classification accuracy [46].

In previous work, ML analysis was successfully applied to psychometric measures [35, 47]. Predictive validity of the SCI was analyzed using logistic regression (LR), random forest (RM), and gradient boosting (GB) algorithms. The scale was proficient in predicting suicidal behavior at one month follow-up (AUROC = 89.4%). The individual items were then ranked based on the ability to predict the outcomes utilizing a chi-square approach [35].

Although ML analysis is useful in obtaining model performance indices like accuracy, or the ratio of the correctly predicted observations to the total observations, it lacks a computational approach for interpreting results [48]. This is especially important when analyzing complex statistical models, such as psychometric models, that can prove difficult to interpret [49]. Shapley Additive Explanation analysis (SHAP) is a mathematical approach designed to aid the interpretation of machine learning outputs. The SHAP framework is essentially a complex ranking system that applies game theory to assess the contributions of individual “features” to the prediction of designated outcomes in complex models, thus providing the means to rank them [48]. In the present study, the features are the SCI-2 items, and the outcome is the concurrent validity to known validated suicidality measures [i.e., the Columbia Suicide Severity Rating Scale (C-SSRS)]. Though already used to interpret complex biological models [50, 51] to our knowledge, SHAP analysis has not yet been employed for the analysis of psychometric scales.

The present study has two aims. Our first aim is to evaluate the feasibility of using the SHAP framework for ML analysis interpretation of the SCI-2 in predicting SI and suicidal preparatory behaviors as measured by the C-SSRS. Our second aim is to rank the predictive performance of the scale’s individual items in order to derive the SCI-2-SF.

Our hypothesis is that our approach will result in meaningful rankings of the SCI-2 items, comprising of all five SCS criteria.

## Materials and methods

### Recruitment

This Study was not preregistered. The International Suicide Prevention and Assessment Risk during COVID (ISPARC) project was a web-based survey study that assessed a variety of psychological features during COVID across 11 different countries (Brazil, Canada, Germany, India, Israel, Poland, Russia, South Korea, Turkey, United States, and Taiwan) from June 2020 until February 2021. Participants were recruited online via posts and paid advertising on social media networks and websites. All participants provided informed written consent via the Qualtrics survey platform before completing a web-based battery of self-report measures. In Turkey, study participants also used paper survey and consent forms, later entered in the online platform manually. Respective Principal Investigators oversaw recruitment and translation and back-translations of all surveys into local languages. Most participating countries did not offer compensation, though South Korean participants received 3000 KRW and United States participants were eligible to enter a raffle for one of thirty $15 gift cards. The study was approved by the relevant Institutional Review Boards/Institutional Ethics Committees in all affiliated institutions.

### Measures

#### Suicide Crisis Inventory-2 (SCI-2) [37]

The SCI-2 is a 61-item self-report questionnaire that assesses SCS severity over the past few days when feeling their worst. Items cover five symptom dimensions, including entrapment, affective disturbance, loss of cognitive control, hyperarousal, and social withdrawal. Responses are rated on a 5-point Likert scale, ranging from 0 (*Not at all*) to 4 (*Extremely*), with selected items recorded such that higher scores are indicative of more severe SCS symptoms. The SCI-2 has demonstrated excellent internal consistency as well as convergent, discriminant, criterion, and predictive validity in past research [21, 52]. Internal consistency in the present study was excellent (α = .98).

#### Columbia-Suicide Severity Rating Scale screener (C-SSRS) [43]

The C-SSRS screener is a 9-item self-report measure that examines SI and suicidal behavior within the past month. Participants respond yes (1) or no (0) to five questions assessing severity of SI, such that total scores range from 0 *(no SI*) to 5 (*SI with intent and a specific plan*). A sixth item assesses preparatory behaviors for suicide, whereas the last three items assess the presence of aborted, interrupted, and actual suicide attempts. The C-SSRS has demonstrated strong reliability and validity in previous research [53, 54]. In this study, items 1 through 5 were used to define our SI outcome, as a yes in any of those five items was coded as 1 (i.e.: presence of SI). Item 6 was used to define the SI with suicide preparatory behaviors (SI-P) outcome.

#### Suicide Crisis Inventory-Short Form (SCI-SF) [34]

The 8-item SCI-SF is an abbreviated version of the SCI that uses its best performing items in the prediction of suicidal thoughts and behaviors. Like the SCI-2, participants respond in relation to the past few days when they have felt their worst [41]. Answers are rated on a 5-point Likert scale, ranging from 0 (*Not at all*) to 4 (*Extremely*), with higher scores reflecting more severe SCS symptoms. When included in the Modular Assessment Risk of Imminent Suicide (MARIS) as one of its patient self-report modules, the SCI-SF demonstrated predictive validity for suicidal thoughts and behaviors at one-month follow-up [55, 56]. In this study, the SCI-SF was not directly administered to participants, but rather, used to assess the validity of the item rankings of each outcome and guide the formation of the SCI-2-SF.

### Statistical analyses

Predictive performance was measured using LR, RF, and GB algorithms. All machine learning algorithms were implemented in Python using a Jupyter notebook with the scikit-learn package. The dataset was loaded as a dataframe using the *pandas* library, and feature importance was measured using the SHAP package. LR was the strongest performer and thus was used for the subsequent feature importance analysis. This analysis was based on the SHAP framework, which combines six separate methods for ranking feature importance for a particular prediction into a single unified technique [48]. This SHAP framework, built upon Shapley values, uses game theory to calculate the marginal contributions of each feature toward the prediction of a particular datapoint, then averages these contributions across all datapoints. This was implemented using the SHAP Python package.

Four-fold cross-validation was used to split the dataset into training and testing samples. This was done in a stratified manner to ensure that each sample retained the same proportion of cases and controls seen in the full dataset. Due to imbalances between the outcomes, the cross-validation process was repeated 1,000 times, and results were aggregated into a final list of the 20 most important features. For each cross-validation iteration, the 20 most important features were stored. Since there were 1,000 iterations, a list was kept of the number of times each feature appeared among the top 20 across the 1,000 iterations. This value, dubbed SHAP Feature Importance (SFI), served as a measure of the relative importance of each feature, and was used to create a ranking list of items based on their individual importance in the prediction of the designated outcome. (see Results section, Table 3).

Our second aim was to build a short form of the SCI-2. To that end, we compared the top 10 items of our rankings to the SCS-SF. The rationale behind this comparison was to select the ranking that resembled most the structure of an already validated measures (the SCI-SF). We chose to compare only the 10 top ranking items in each item listing, because SFI dropped to below half its starting value after the 10^th^ position in the rankings (Table 3). We ultimately selected our results regarding the SI-P outcome instead of those considering only SI as the outcome, as the former resembled most the SCI-SF. We then used the top two ranking items for each SCS criterion (i.e., entrapment, affective disturbance, loss of cognitive control, hyperarousal, social withdrawal) to build the SCI-2-SF. To assess social withdrawal, lacking in our SI-P results, we utilized the top-ranking social withdrawal item from the SI results.

Materials for this study are not available.

## Results

### Sample characteristics

The sample was composed of (10,357) adults recruited from 11 participating countries (Brazil: *n* = 127; Canada: *n* = 65; Germany: *n* = 532; India: *n* = 302; Israel: *n* = 195; Poland: *n* = 309; Russia: *n* = 561; South Korea: *n* = 1,043; Taiwan: *n* = 4846; Turkey: *n* = 424; and the United States: *n* = 1,970) between June 2020 and February 2021. Inclusion criteria were being a legal adult and residing in one of the 11 countries during the pandemic. No specific exclusion criteria were applied. Detailed sociodemographic information, stratified by country, is presented in Table 1. Most of the participants self-identified as cisgender women, were single/never married, and had completed a degree at a 4-year university or college.

**Table 1:** Sociodemographic characteristics of all participants, stratified by country.

	Brazil	Canada	Germany	India	Israel	Poland	Russia	South Korea	Taiwan	Turkey	United States
**Age (Mean/SD)**	34.6 (13.6)	34.2 (12.9)	40.1 (15.5)	43.5 (17.9)	36.8 (17.2)	32.0 (13.4)	26.9 (12.7)	30.3 (8.2)	37.55 (10.83)	33.4 (9.5)	26.7 (8.48)
**Gender (%)**											
Male	24 (18.9)	19 (29.2)	131 (24.6)	140 (46.4)	37 (19.0)	40 (12.9)	135 (24.1)	290 (27.8)	822 (16.8)	175 (41.3)	213 (10.8)
Female	103 (81.1)	43 (66.2)	397 (74.6)	162 (53.6)	158 (81.0)	252 (81.6)	408 (72.7)	753 (72.2)	4038 (82.6)	249 (58.7)	1722 (87.4)
Trans Male	0 (0.0)	0 (0.0)	--	0 (0.0)	0 (0.0)	0 (0.0)	1 (0.2)	--	4 (0.1)	--	6 (0.3)
Trans Female	0 (0.0)	0 (0.0)	--	0 (0.0)	0 (0.0)	3 (1.0)	4 (0.7)	--	0 (0.0)	--	6 (0.3)
Non-Binary	0 (0.0)	2 (3.1)	3 (0.6)	0 (0.0)	0 (0.0)	11 (3.6)	4 (0.7)	--	2 (0.0)	--	18 (0.9)
Not Sure	0 (0.0)	0 (0.0)	--	0 (0.0)	0 (0.0)	2 (0.6)	4 (0.7)	--	1 (0.0)	--	2 (0.1)
Decline to State	0 (0.0)	1 (1.5)	1 (0.2)	0 (0.0)	0 (0.0)	1 (0.3)	5 (0.9)	--	21 (0.4)	--	3 (0.2)
**Marital Status (%)**									—		
Never Married	34 (26.8)	31 (47.7)	117 (22.0)	83 (27.5)	63 (32.3)	118 (38.2)	311 (55.4)	722 (69.2)	1997 (40.9)	195 (46.0)	1059 (53.8)
Married	48 (37.8)	14 (21.5)	177 (33.3)	190 (62.9)	63 (32.3)	64 (20.7)	86 (15.3)	298 (28.6)	2224 (45.5)	211 (49.8)	225 (11.4)
Separated	1 (0.8)	1 (1.5)	8 (1.5)	1 (0.3)	1 (0.5)	2 (0.6)	19 (3.4)	1 (0.1)	13 (0.3)	“Other”: 18 (4.2)	17 (0.9)
Divorced	4 (3.1)	3 (4.6)	24 (4.5)	4 (1.3)	8 (4.1)	20 (6.5)	19 (3.4)	6 (0.6)	170 (3.5)		40 (2.0)
Widowed	25 (19.7)	0 (0.0)	7 (1.3)	6 (2.0)	2 (1.0)	9 (2.9)	6 (1.1)	1 (0.1)	31 (0.6)		5 (0.3)
Dating R/S	13 (10.2)	12 (18.5)	115 (21.6)	14 (4.6)	46 (23.6)	87 (28.2)	83 (14.8)	--	305 (6.2)		449 (22.8)
Cohabitating	2 (1.6)	4 (6.2)	84 (15.8)	4 (1.3)	12 (6.2)	9 (2.9)	37 (6.6)	15 (1.4)	148 (3.0)		175 (8.9)
**Education (%)**											
Less than HS	1 (0.8)	5 (9.2)	22 (4.2)	0 (0.0)	1 (0.5)	3 (1.0)	9 (1.6)	2 (0.2)	8 (0.2)	56 (13.2)	4 (0.2)
HS or Equivalent	7 (5.5)	10 (15.4)	52 (9.8)	19 (6.3)	17 (8.7)	20 (6.5)	250 (44.6)	80 (7.7)	201 (4.1)	87 (20.5)	78 (4.0)
2-Year College	2 (1.6)	10 (15.4)	123 (23.1)	11 (3.6)	7 (3.6)	14 (4.5)	118 (21.0)	94 (9.0)	—	47 (11.1)	162 (8.2)
Some College	46 (36.2)	--	--	20 (6.6)	77 (39.5)	123 (39.8)	49 (8.7)	--	—	--	404 (20.5)
4-year College	56 (44.1)	28 (43.1)	98 (18.4)	146 (48.3)	33 (16.9)	36 (11.7)	60 (10.7)	624 (59.8)	3668 (75.0)	155 (36.6)	977 (49.6)
Master’s Degree	11 (8.7)	8 (12.3)	120 (22.6)	91 (30.1)	44 (22.6)	104 (33.7)	56 (10.0)	213 (20.4)	1011 (20.7)	59 (13.9)	286 (14.5)
Doctoral Degree	4 (3.1)	3 (4.6)	117 (22.0)	15 (5.0)	16 (8.2)	9 (2.9)	19 (3.4)	30 (2.9)	—	20 (4.7)	59 (3.0)

*Note*. Means and standard deviations are presented for age. Raw number and proportions are presented for all other categories. “—”means that the response option was not provided in that country. “Dating R/S” = In a Dating Relationship. HS = High School.

Responses to suicide-related outcomes (past-month SI and SI-P) indicated that there were 8,378 negative controls ("0") and 1,979 positive cases ("1") for SI, meaning that only 19.11% of participants expressed SI within the previous month, and 10,126 negative controls ("0") and 241 positive cases ("1") for SI-P, meaning that only 2.23% of participants had made any preparations for a suicide attempt within the previous month.

### Machine learning algorithms

ML performance indices are displayed in detail in Table 2. For a detailed explanation of the different performance indices, which goes beyond the current aims of the paper, please see the work of Parghi and colleagues in 2021 [35]. Of the three machine learning algorithms, LR outperformed GB and RF when considering both outcomes. For this reason, we chose to describe and evaluate only LR results. The difference in AUPRC between the SI and the SI-P outcomes is likely due to the imbalance between cases and controls when considering the SI-P outcome (2.2% of cases) [57].

**Table 2:** Machine learning algorithm performance indices.

Algorithm/Outcome	Accuracy	AUROC	Precision	Recall	Balanced Accuracy	AUPRC
**LR—SI**	0.855	0.856	0.677	0.461	0.704	0.633
**LR–SI-P**	0.977	0.824	0.369	0.026	0.512	0.144
**GB—SI**	0.847	0.835	0.638	0.462	0.700	0.600
**GB–SI-P**	0.977	0.772	0.356	0.028	0.513	0.106
**RF–SI**	0.851	0.856	0.682	0.414	0.684	0.616
**RF–SI-P**	0.977	0.803	0.197	0.006	0.503	0.132

*Note*. AUROC: Area Under Receiver Operating Characteristic; AUPRC: Area Under Precision-Recall Curve; LR: Logistic Regression; GB: Gradient Boosting; RF: Random Forest.

### SHAP feature importance rankings

The SHAP ranking of the top 15 SCS-2 items for both outcomes is presented in Table 3. Regarding the SI outcome, the top 10 ranking items include every SCS criterion. The best performing item, SCI-12 (“Did you feel hopeless?”) is associated with the entrapment criterion. The next best performing items are associated with the hyperarousal criterion, then the affective disturbance criterion, and lastly the social withdrawal and loss of cognitive control criteria in the 9^th^ and 10^th^ positions, respectively.

**Table 3:** Top ranking items for suicidal ideation (SI) and preparations (SI-P) outcomes compared to the SCI-SF.

R^a^	SI SFI^b^	SI Items	SI SCS Criteria	SI-P SFI^b^	SI-P Items	SI-P SCS criteria
1	4000	SCI-12—Did you feel hopeless?	A. Entrapment	3972	SCI-57—Did you enjoy being with your family or close friends?	B1. Affective Disturbance
2	4000	SCI-60 –Did you feel you wanted to crawl out of your skin?	B3. Hyperarousal	3260	SCI-11—Did you feel that it was hard for you to stop worrying?	B2. Loss of Cognitive Control
3	3999	SCI-59—Did you feel so restless you could not sit still?	B3. Hyperarousal	2914	SCI-41—Did you feel that your emotional pain was unbearable?	B1. Affective Disturbance
4	3977	SCI-38—Did you feel a sense of inner pain that had to be stopped?	B1. Affective Disturbance	2678	SCI-7—Did you have a sense of inner pain that was too much to bear?	B1. Affective Disturbance
5	3918	SCI-4—Did you feel there was no exit?	A. Entrapment	2655	SCI-48—Did you feel that the urge to escape the pain was very hard to control?	B1. Affective Disturbance
6	3901	SCI-51—Did you feel any unusually intense or deep negative feelings or mood swings directed towards yourself?	B1. Affective Disturbance	2224	SCI-4—Did you feel there was no exit?	A. Entrapment
7	3841	SCI-72—Did you feel easily annoyed or irritated?	B3. Hyperarousal	1833	SCI-12 - Did you feel hopeless?	A. Entrapment
8	3306	SCI-57r –Did you enjoy being with your family or close friends? R	B1. Affective Disturbance	1749	SCI-1—Did you wake up from sleep tired and not refreshed?	B3. Hyperarousal
9	2339	SCI-74—Did you feel you did not open up to members of your family/friends?	B4. Social Withdrawal	1638	SCI-14—Did you have a decreased ability to think, concentrate or make decisions, due to too many thoughts?	B2. Loss of Cognitive Control
10	1870	SCI-3—Did you have many thoughts in your head?	B2. Loss of cognitive control	1609	SCI-38—Did you feel a sense of inner pain that had to be stopped?	B1. Affective Disturbance
11	1807	SCI-41—Did you feel that your emotional pain was unbearable?	B1. Affective Disturbance	1601	SCI-61—Did you feel so stirred up inside you wanted to scream?	B3. Hyperarousal
12	1192	SCI-13—Did you feel that there was no way out?	A. Entrapment	1464	SCI-34—Did you feel that ideas kept turning over and over in your mind and they wouldn’t go away?	B2. Loss of Cognitive Control
13	580	SCI-54—Did you feel dissatisfied or bored with everything?	B1. Affective Disturbance	1398	SCI-47—Did you feel like you were getting a headache from too many thoughts in your head?	B2. Loss of Cognitive Control
14	534	SCI-45—Did you feel pressure in your head from thinking too much?	B2. Loss of cognitive control	1217	SCI-35—Did you feel doomed?	A. Entrapment
15	235	SCI-7—Did you have a sense of inner pain that was too much to bear?	B1. Affective Disturbance	1142	SCI-24—Did you feel your thoughts were racing?	B2. Loss of Cognitive Control

Note.

^a^R: rank, referred to SI and SI-P results

^b^SFI: SHAP Feature Importance; SCS: Suicide Crisis Syndrome; SCI-SF: Suicide Crisis Inventory Short Form; SCI: Suicide Crisis Inventory.

When considering SI-P as the outcome, the top 10 ranked items include every SCS criterion except social withdrawal. The best performing item, SCI-12 (“Did you feel hopeless?”), is associated with the entrapment criterion, followed by items in the loss of cognitive control, entrapment, and hyperarousal criteria. Items associated with the affective disturbance criterion are most prevalent (4^th^, 6^th^, 8^th^, 11^th^, 13^th^, 15^th^), whereas the social withdrawal criterion is represented only by one top 10 item.

### SHAP ranking compared to SCI-SF

**Fig 1:**
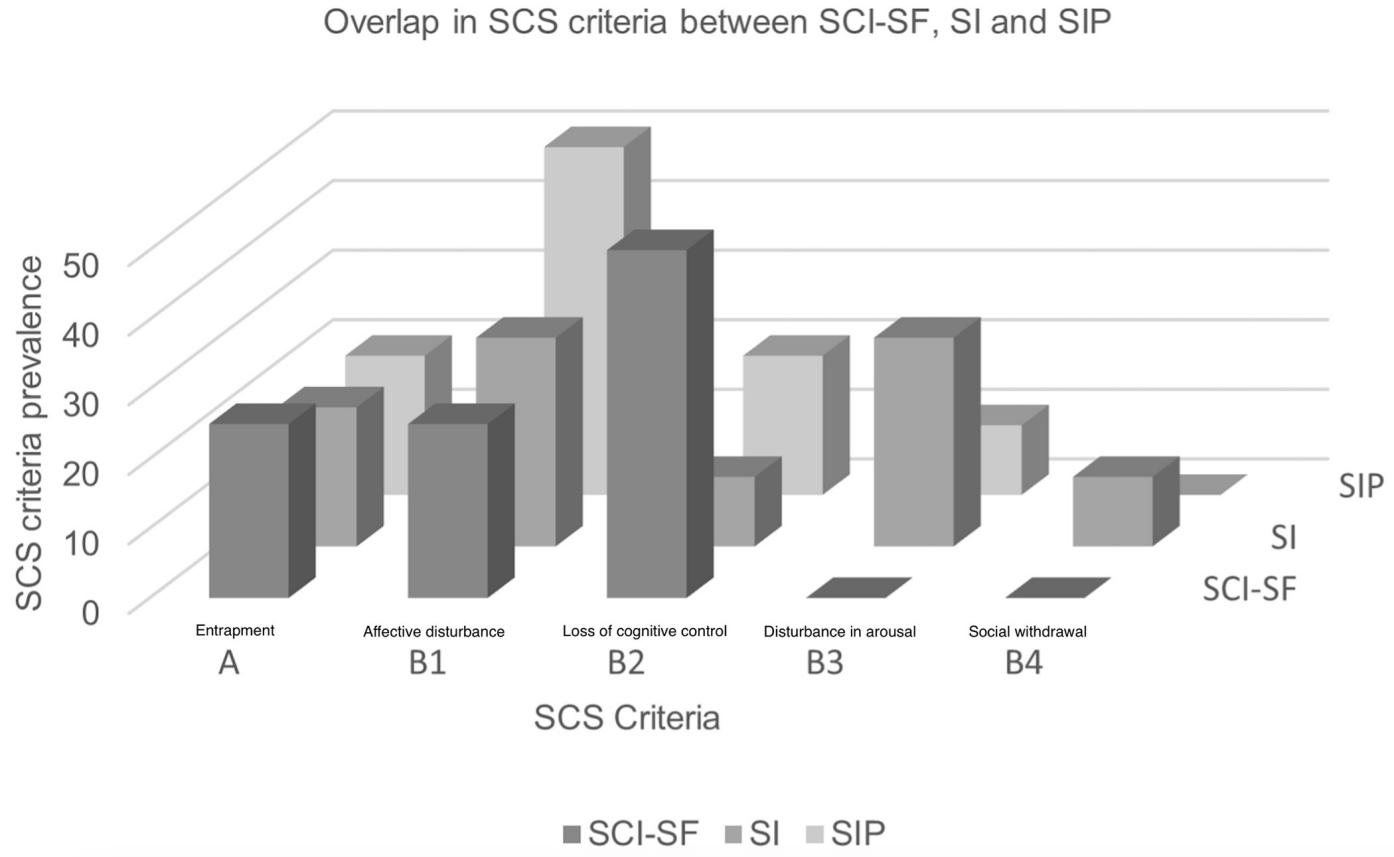
Column graph depicting the overlap in SCS criteria between the SCI-SF and the SI-P and SI results. *Note*. The prevalence of SCS criteria (y-axis) was measured as the percentage of items in each scale/ranking belonging to a particular criterion. A: Entrapment, B1: Affective Disturbance, B2: Loss of Cognitive Control, B3: Hyperarousal, B4: Social Withdrawal.

In the comparison between the SCI-SF and our results, summarized in Table 3 and Fig 1, we evaluated the grade of overlap between the former and the latter items’ SCS criteria distribution. The ranking of each individual item was also considered. This was done with the aim to estimate the face validity of our results when compared to a valid and reliable existing measure, and in doing so, select the most appropriate item ranking with which to build the SCI-2-SF.

Specifically, the SCI-SF (Table 3) contains items that cover three of the five SCS criteria, excluding social withdrawal and hyperarousal. Loss of cognitive control is the most frequently represented with four of eight items (SCI-2, 8, 26, and 47), followed by the entrapment and affective disturbance criteria with two of eight items (SCI-4 and 30; SC-I5 and 37). Considering both the ranking and criteria distribution of the top 10 ranking items, the SCI-SF is most similar to our results using SI-P as an outcome. The most prevalent criterion is affective disturbance followed by the loss of cognitive control and entrapment criteria. The least represented criterion is hyperarousal. When considering our results using only SI as the outcome, on the other hand, the 10 top ranking items differ substantially from the SCI-SF. Hyperarousal is the most represented and well ranking criterion, whereas the least represented and poorest ranking criteria are social withdrawal and loss of cognitive control.

### SCI-2-SF

The resultant SCI-2-SF consists of the top two ranking items for each SCS criterion/factor (only one for social withdrawal), namely a total of nine items that cover all SCS symptoms. The final SCS-2-SF assessment tool, which covers the whole span of SCS domains, is presented in Table 4. Its best performing items belong to the Affective Disturbance (SCI57: “Did you enjoy being with your family or close friends?” [reverse coded]), Loss of Cognitive Control (SCI11: “Did you feel that it was hard for you to stop worrying?”) and Entrapment (SCI4: “Did you feel there was no exit?") criteria.

**Table 4:** Items included in the final SCI-2-SF.

Criteria	Ranking	Items
A. Entrapment	1	SCI-12—Did you feel hopeless?
	5	SCI-4—Did you feel there was no exit?
B1. Affective Disturbance	4	SCI-38—Did you feel a sense of inner pain that had to be stopped?
	6	SCI-51—Did you feel any unusually intense or deep negative feelings or mood swings
B2. Loss of cognitive control	10	SCI-3—Did you have many thoughts in your head?
	14	SCI-14—Did you have a decreased ability to think, concentrate or make decisions, due to too many thoughts?
B3. Hyperarousal	2	SCI-60 –Did you feel you wanted to crawl out of your skin?
	3	SCI-59—Did you feel so restless you could not sit still?
B4. Social Withdrawal	9	SCI-74—Did you feel you did not open up to members of your family/friends?

## Discussion

This SHAP analysis of the SCI-2 self-report scale resulted in meaningful rankings of the SCI-2 individual items, since its highest-ranking items included all five SCS criteria [39, 58]. Our findings additionally indicate that the SHAP method can be applied successfully to ML analysis of psychometric measures. To our knowledge, this is the first successful application of the SHAP framework to the analysis of suicide-related measures or of any psychometric assessment tool. As expected, the SCI-2 rankings overlapped significantly with the SCI-SF and included additional items associated with overarousal and social withdrawal. Similarly, the SCI-2-SF overlapped greatly with the SCI-SF, while also including items associated with overarousal and social withdrawal.

Of the two outcomes we utilized in the analyses, the one that included suicide without preparatory actions produced results that most resembled the structure of the SCI-SF, compared to those derived by the SI-P outcome. As a suicide preparatory behavior is, by definition, any act or preparation towards an imminent premeditated suicide attempt [16], our results appear in line with research that describes the SCS as a construct especially proficient in predicting short-term suicidal behavior [37, 30].

The SCI-2-SF consists of the top two ranking items for each SCS criterion/factor (only one for social withdrawal) resulting in nine items that cover all SCS symptoms. This approach is consistent with previous work; Bloch-Elkouby and colleagues (2021) used factor analysis to show that the SCS is composed of five distinct factors that all contribute to its predictive validity for suicidal thoughts and behaviors [59]. Yaseen and colleagues (2019) demonstrated that the SCS has superior predictive validity when both criteria A and B are met simultaneously [37]. Additionally, Bafna and colleagues (2022) illustrated that both 1-factor and 5-factor configurations of the SCS 5-factor model showed good model fit in a large clinical sample [60].

Although the implementation of the SCS in clinical settings is still in early phases, preliminary data submitted for publication from an independent group of Northshore Hospital in Chicago, Illinois clearly indicates that the SCS altered how ER clinicians treated suicidal patients. When clinicians used an abbreviated form of the SCS checklist, patients that screened positive for the SCS were 66 times more likely to be admitted into the inpatient unit compared to those who screened negative. Additionally, patients in the ER who exhibited SI as their main complaint in the absence of the SCS were 80% more likely to be discharged than those without SI, thereby demonstrating how the implementation of the SCS could help identify malingering or low-risk individuals. Although no items in the SCI-2 (or in the SCI-2-SF) explicitly inquire about SI or behaviors, our approach was highly accurate at assessing concurrent preparatory suicidal behavior. This could result in the detection of individuals who consciously or unconsciously hide their suicidal thoughts but might otherwise remain undetected. Furthermore, deeming a patient as high-risk could lead to his/her/their subsequent disclosure of previously concealed SI [61], which could foster the therapeutic alliance.

Further studies are necessary to determine an ideal SCS-SF-2 score threshold that optimizes sensitivity and specificity for an SCS diagnosis. Thus, the current version of the SCI-SF-2 serves as a tool to assess SCS severity in a dimensional fashion and could be used in conjunction with other instruments capable of a categorical SCS diagnosis, critical in clinical decision-making, such as the SCS checklist [33], and other traditional methods like the C-SSRS or suicide-related history to achieve an optimal assessment of suicide risk.

Clinical work and research in the mental health field is largely based on a categorical diagnostic system, such as the DSM–5 and ICD–10. Although certainly useful, this approach has various limitations, such as in the definition of exhaustive and mutually exclusive diagnostic categories [62–65]. These limitations can often result in significant heterogeneity between diagnoses, and many cases tend to be categorized as “not otherwise specified” [66]. Therefore, many members of the scientific community are striving for a dimensional approach to psychiatric nosology and psychopathology, with models like the Hierarchical Taxonomy of Psychopathology [67] and the Research Domain Criteria [68]. At present, clinicians use the categorical approach, whereas researchers have been focusing on and arguing for a dimensional diagnosis. Should the latter approach prevail, the SCI-SF-2 would be an excellent practical tool, as it is informative on multiple psychopathological dimensions and potentially useful to clinicians in diverse scenarios.

### Limitations and future directions

Our study presents several limitations that are worth noting. First, the cross-sectional nature of the study impedes any inference of temporality and, in so doing, any measure of predictive validity. Repeating the analyses in a longitudinal, prospective study could help to assess the SCI-2-SF capacity to predict suicidal thoughts and behaviors over time. Second, our study examined the ability of the SCI-2 to assess concurrent measures of SI and suicidal preparatory acts, although the SCS is more proficient at predicting suicidal behavior than ideation according to previous work [30, 37]. However, the higher incidence of the designated outcomes was deemed necessary due to the relative rarity of suicide attempts in the general population [69] and to the high number of cases, or events per variable, needed to achieve reliable results in ML and SHAP analyses [70, 71]. Despite these precautions, the number of positive cases for suicidal preparatory behaviors was only 2.23% of the negative controls (241 vs 10,126). Though unavoidable due to the nature of suicidal phenomena and the use of a convenience community-based sample, repeating the study with a less unbalanced dataset could potentially lead to more accurate and reliable results. The data fromthis convenience sample was collected during the pandemic, which may have affected our results, considering that these results may differ in times of collective stress and between various high-risk groups. Finally, the C-SSRS utilized a hierarchal structure, which is error prone and might have introduces some bias to our study [72].

## Conclusion

We successfully applied predictive ML analyses and SHAP feature rankings to the SCI-2. We subsequently developed a brief assessment tool, the SCI-SF-2, which we anticipate achieving good predictive validity in future prospective studies. Our results add to accumulating research that demonstrates how the SCS and its assessment tools show concurrent, incremental, and predictive validity for SI and suicidal behavior [33, 35–37, 59, 73], in proving the potential clinical utility of the SCS in the assessment of individuals at high risk of near-term STBs. Due to its brevity, self-report nature, and ease of use, we believe that the SCS-2-SF could be easily introduced in clinicians’ workflow to assess SCS severity.

## Supporting information

S1 File (XLSX)
